# Telemedicine for cardiometabolic disease control in Latin America and the Caribbean: A systematic review and meta-analysis

**DOI:** 10.1016/j.pmedr.2026.103633

**Published:** 2026-09-23

**Authors:** Kevin Saldarriaga-Bedoya, Sergio César Ruiz-Jiménez, Santiago José Sánchez-Rodríguez, Gladys Rocío Ariza-Sosa, Sjoukje van der Werf, Erik Buskens, Maarten Lahr

**Affiliations:** a Department of Epidemiology, *University Medical Center Groningen,* University of Groningen*,* the Netherlands; b Hospital Alma Máter de Antioquia, Medellín, Colombia; c Facultad de Medicina, Universidad de Antioquia, Medellín, Colombia; d Medical Library, *University Medical Center Groningen,* University of Groningen*,* the Netherlands

**Keywords:** Telemedicine, Cardiometabolic disease, Type 2 diabetes mellitus, Hypertension, Latin America and the Caribbean, Primary care

## Abstract

**Objective:**

To evaluate the effectiveness of telemedicine for cardiometabolic disease control in Latin America and the Caribbean (LAC).

**Methods:**

We systematically searched international and regional databases from inception to 17 December 2025 for telemedicine interventions in adults with type 2 diabetes, hypertension, or dyslipidaemia in LAC. Risk of bias and certainty of evidence were assessed using validated tools and GRADE, respectively. Random-effects meta-analyses included clinically comparable randomised trials.

**Results:**

Twenty-two studies from 10 countries were included (11 randomised). Meta-analyses included seven trials (935 participants) for HbA1c, four (451) for systolic blood pressure, and three (280) for diastolic blood pressure. Pooled estimates favoured telemedicine for HbA1c (MD −0.62%, 95% CI −1.02, −0.22; low certainty), systolic blood pressure (MD −6.60 mmHg, 95% CI −9.49, −3.71; high certainty), and diastolic blood pressure (MD −4.25 mmHg, 95% CI −6.53, −1.98; moderate certainty). HbA1c heterogeneity was substantial (I^2^ = 66%), and its prediction interval crossed the null (−1.77%, 0.56%).

**Conclusions:**

Selected telemedicine interventions may improve intermediate cardiometabolic outcomes in specific LAC settings. However, limited and heterogeneous evidence, variable certainty, and insufficient evidence on long-term effectiveness, scalability, cost-effectiveness, and equity preclude broad implementation recommendations.

**PROSPERO:**

CRD420251234521.

## Highlights

• Selected telemedicine interventions may improve cardiometabolic outcomes in specific Latin American and Caribbean settings.
• Pooled estimates favoured telemedicine for glycated haemoglobin and blood pressure.
• An exploratory pattern suggested greater benefits with some clinician-intensive interventions, although this was not formally compared.
• Evidence for equity, costs and long-term outcomes remains limited.
• More high-quality trials are needed across diverse healthcare settings.

## 1 Introduction

Cardiometabolic diseases, including type 2 diabetes mellitus (T2DM), hypertension, and dyslipidaemia, are major causes of morbidity and mortality in Latin America and the Caribbean (LAC). In 2020, the age-standardised prevalence of hypertension approached 40% among adults (Pan American Health Organization, 2023), while diabetes remains substantially underdiagnosed in South and Central America (International Diabetes Federation, 2025). Cardiovascular disease causes approximately 1.6 million deaths annually in the Americas (World Health Organization, 2023), and cardiometabolic control remains suboptimal (Pan American Health Organization, 2023; World Health Organization, 2023). The COVID-19 pandemic disrupted chronic-disease care across LAC. In a PAHO assessment of 28 member states, outpatient non-communicable disease services were partially interrupted in 64% of countries, and two-thirds replaced some face-to-face consultations with telemedicine (Pan American Health Organization, 2020). Telemedicine may support continuity and access where geographic and health-system barriers constrain chronic-disease management, although the evidence remains limited and heterogeneous.

Previous systematic reviews and expert recommendations have reported modest improvements in glycated haemoglobin (approximately 0·3–0·6%) and systolic blood pressure (3–6 mmHg), but most evidence originates from high-income countries, focuses on individual cardiometabolic conditions or specific telemedicine modalities, and varies considerably in intervention design, intensity, and setting (Kebede and Pischke, 2019; Omboni et al., 2020; Hoffer-Hawlik et al., 2021). Whether these findings are applicable to Latin America and the Caribbean remains uncertain. The effectiveness and implementation of telemedicine may differ in the region because of differences in health-system organisation, availability and distribution of healthcare professionals, financing of remote care, digital infrastructure and connectivity, access to digital technologies, and socioeconomic and digital-literacy barriers. These contextual factors may influence telemedicine access, uptake, continuity, clinician involvement, and treatment effectiveness. Importantly, previous reviews have not quantified telemedicine effectiveness for cardiometabolic disease in LAC, have often focused on individual conditions or modalities, and may have underrepresented Spanish- and Portuguese-language publications and regional grey literature (Hoffer-Hawlik et al., 2021; Ogungbe et al., 2025). Consequently, there remains no comprehensive region-specific synthesis establishing the magnitude and certainty of the available evidence across type 2 diabetes mellitus, hypertension, and dyslipidaemia in Latin American and Caribbean healthcare settings. This review addressed that gap.

Telemedicine is not a uniform intervention: strategies range from telephone or message-based follow-up and mobile applications to remote monitoring, clinician-led medication adjustment, and multidisciplinary management. Differences in intervention content, intensity, contact frequency, and clinician involvement may influence effects and determine which studies are comparable for quantitative synthesis.

This systematic review aimed to determine the effectiveness of diverse telemedicine strategies in improving clinical indicators of cardiometabolic disease control, including glycated haemoglobin, systolic and diastolic blood pressure, and lipid profiles, among adults with type 2 diabetes mellitus, hypertension, or dyslipidaemia in Latin America and the Caribbean. Secondary objectives were to assess medication adherence, patient satisfaction and engagement, and healthcare service utilisation; explore social, economic, gender-related, and other structural inequities that may influence access to, uptake of, or outcomes associated with telemedicine; and characterise the types, modalities, and delivery components of telemedicine interventions implemented across the region.

## 2 Methods

This review followed PRISMA 2020 (Page et al., 2021) (Supplementary S2 File) and was prospectively registered in PROSPERO (CRD420251234521). There were no deviations from the prespecified population, scope, interventions, outcomes, or risk-of-bias and certainty assessments. The protocol prespecified narrative synthesis, random-effects meta-analysis when studies were sufficiently homogeneous, heterogeneity assessment using I^2^, subgroup analyses by disease, telemedicine modality, and country or subregion, and sensitivity analyses. Statistical procedures were further specified during analysis, including the variance estimator, Hartung-Knapp-Sidik-Jonkman adjustment, prediction intervals, and leave-one-out analyses. Prespecified subgroup analyses were not performed because fewer than ten clinically comparable randomised trials were available for any pooled outcome.

### 2.1 Data sources and search strategy

We systematically searched PubMed, Embase, Web of Science Core Collection, Scopus, Cochrane CENTRAL, and LILACS from inception to 17 December 2025. Regional bibliographic sources included BVS, SciELO, RedALyC, and Latindex. Grey-literature and other sources included PAHO/WHO IRIS, institutional repositories, ProQuest Dissertations & Theses Global, ClinicalTrials.gov, and the WHO International Clinical Trials Registry Platform, supplemented by citation tracking and expert consultation. The search strategy was developed and reviewed with a medical information specialist (SvdW), who led the methodology. The search strategy did not undergo PRESS peer review. Searches combined controlled vocabulary and free-text terms for cardiometabolic conditions, telemedicine and digital health, study design, and Latin America and the Caribbean, without language, date, or publication-status restrictions (Supplementary S1 File).

### 2.2 Eligibility criteria and study selection

We included adults (≥18 years) with T2DM, hypertension, or dyslipidaemia receiving primary or secondary care in LAC. Geographical eligibility followed the prespecified PROSPERO scope (CRD420251234521), including English-, French-, and Dutch-speaking Caribbean territories; eligibility was geographical, with no language restrictions. Eligible telemedicine interventions used telecommunications or digital technologies for clinical evaluation, monitoring, treatment, or follow-up, with in-person care, alternative telemedicine, usual care, or baseline measurements as comparators. Outcomes included glycaemic control, blood pressure, lipid parameters, healthcare utilisation, medication adherence, engagement, satisfaction, equity, and implementation. Eligible designs included randomised, quasi-experimental and other non-randomised interventional, cohort, retrospective observational, and pre-post studies; case reports, narrative reviews, conference abstracts without sufficient data, and qualitative-only studies were excluded.

Records were deduplicated with DedupEndNote (Lobbestael, 2026) and independently screened in Rayyan (Ouzzani et al., 2016) by two reviewers (KSB, SCRJ), with substantial agreement (title/abstract kappa = 0·77; full-text kappa = 0·63); disagreements were resolved by discussion or a third reviewer (ML) (Supplementary S3 File; Fig. 1).

**Fig. 1:**
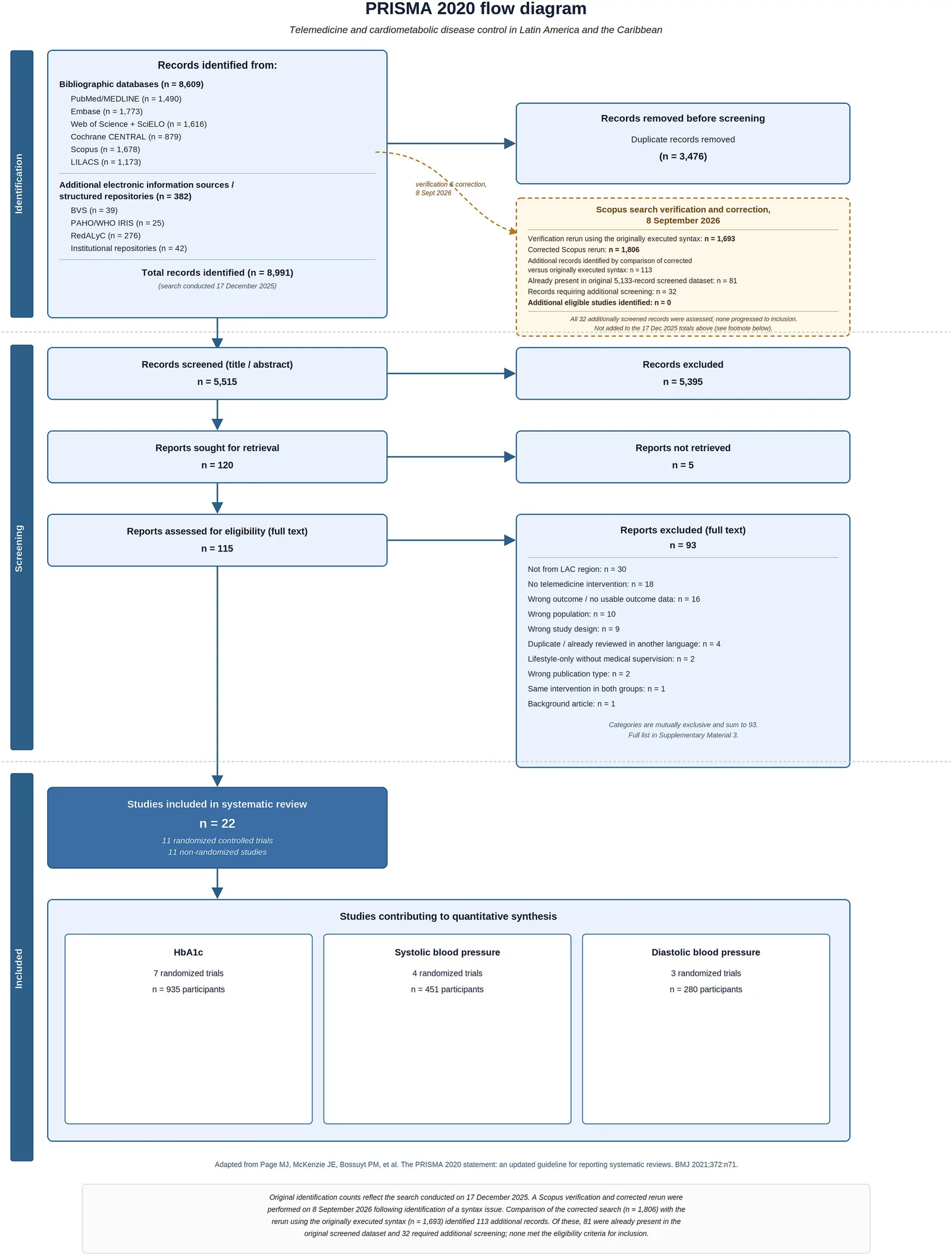
PRISMA 2020 flow diagram of study identification, screening, eligibility, and inclusion for telemedicine interventions in adults with type 2 diabetes, hypertension, or dyslipidaemia in Latin America and the Caribbean, from database inception to 17 December 2025. PRISMA 2020 flow diagram. Counts derived from database searches, DedupEndNote deduplication output, and Rayyan title/abstract and full-text screening exports. No additional eligible records were identified through backward citation tracking, forward citation tracking, or expert consultation.

### 2.3 Data extraction and quality assessment

Data were independently extracted by two reviewers (KSB, SJSR) using a piloted standardised form (Supplementary S4 File), with missing dispersion statistics calculated or requested from authors. Multiple reports were identified by comparing study characteristics, recruitment periods, and participant samples; the most complete and recent report was the primary source, with related reports used for complementary information without double-counting participants.

When multiple estimates were reported, we prioritised the prespecified primary between-group analysis at the longest eligible follow-up, using intention-to-treat estimates and adjusted effects when available; otherwise, estimates were derived from the reported group-level data. When outcomes were reported at multiple analytical levels or as both primary and secondary endpoints, we prioritised estimates corresponding to the study's prespecified primary analysis when available. Adjustment variables varied across studies according to their prespecified analytical models, and adjusted and unadjusted estimates were therefore not assumed to be methodologically equivalent. Final values and change-from-baseline estimates were combined when reported on the same scale; HbA1c was harmonised as percentage points and blood pressure as mmHg, with effect directions standardised so negative values indicated greater reductions with telemedicine.

Primary outcomes were HbA1c, systolic and diastolic blood pressure, and lipid profiles. Secondary outcomes were medication adherence, satisfaction, engagement, healthcare utilisation, feasibility, cost-effectiveness, and equity-related outcomes, extracted using PROGRESS-Plus domains (O'Neill et al., 2014; Karran et al., 2023) and synthesised narratively because of inconsistent reporting. Risk of bias was assessed independently by two reviewers (KSB, SJSR) using RoB 2 for randomised trials (Sterne et al., 2019) and ROBINS-I for non-randomised studies (Sterne et al., 2016) (Supplementary S5 File), and certainty of evidence was assessed using GRADE (Supplementary S6 File) (Schünemann et al., 2013).

### 2.4 Data synthesis and Meta-analysis

Random-effects meta-analysis was performed when at least three randomised trials reporting the same outcome were considered clinically and methodologically comparable by two reviewers, considering target condition, intervention content and intensity, clinician involvement, comparator, follow-up duration, and outcome definition and measurement; disagreements were resolved by a third reviewer. Outcomes were synthesised narratively when fewer than three comparable randomised trials were available, outcome definitions were incompatible, clinical heterogeneity precluded pooling, or sufficient quantitative data were unavailable. Models used inverse-variance weighting with restricted maximum likelihood and Hartung-Knapp-Sidik-Jonkman adjustment, with separate analyses for HbA1c, systolic, and diastolic blood pressure (R Core Team, 2025; Viechtbauer, 2010; Balduzzi et al., 2019). Non-randomised and uncontrolled pre-post studies were not pooled given their susceptibility to confounding and regression to the mean. Heterogeneity was assessed using Cochran's Q, I^2^, and τ^2^, with 95% prediction intervals calculated; Prespecified subgroup analyses by disease, telemedicine modality, and country or subregion were not performed because fewer than ten clinically comparable randomised trials were available for any pooled outcome; meta-regression was not prespecified and was not undertaken. Sensitivity analyses included leave-one-out analysis and alternative τ^2^ estimation. Analyses were performed using R (version 4.4.1; R Foundation for Statistical Computing, Vienna, Austria) with the meta and metafor packages (R Core Team, 2025; Viechtbauer, 2010; Balduzzi et al., 2019).

## 3 Results

The search identified 8991 records (8609 from bibliographic databases and 382 from additional sources); after removing 3476 duplicates, 5515 records were screened and 5395 excluded. Of 120 reports sought, 115 full texts were assessed and five could not be retrieved; 93 were excluded after full-text review. Citation tracking, expert consultation, and the corrective Scopus search identified no additional eligible studies. Overall, 22 studies met the inclusion criteria (Fig. 1).

The 22 studies originated from ten LAC countries, most commonly Brazil and Mexico (five each) and Peru (three), with 15 (68%) published after 2020 (Table 1). Eleven were randomised trials and 11 non-randomised interventional, quasi-experimental, or observational studies; sample sizes ranged from 38 to 3608 participants and follow-up from 1 week to 49 months. Thirteen studies focused on T2DM, eight on hypertension, and two on mixed cardiometabolic populations. Comparators included usual or structured in-person care and baseline measurements in uncontrolled studies. Interventions included telephone follow-up, mobile applications or messaging, telemonitoring, video consultations, and hybrid care, sometimes incorporating medication titration or clinician-led treatment modification. Equity-related characteristics were inconsistently reported.

**Table 1:** Characteristics of 22 studies evaluating telemedicine interventions for cardiometabolic disease control in adults across Latin America and the Caribbean, published between 2010 and 2025.

Author (year), ref.	Country	Design	n	Population	Intervention	Comparator	Primary outcome result
Vázquez-Martínez (2025) ^S1^	Mexico	Retrospective cohort	287	Adults with T2DM, primary care	Telephone calls (monthly), 12 months	Pre-intervention baseline	HbA1c 6·7% → 6·5%; no between-group comparison
Piette (2011) ^S2^	Honduras	Single-arm pre-post	85	Adults with T2DM, primary care	IVR cloud-based programme, 6 weeks	Pre-intervention baseline	HbA1c −1·1% in adherent patients; OR 11·9 (95% CI 1·2, 115·4)
Urbina-Arronte (2025) ^S3^	Mexico	Pre-post hybrid programme	104	Adults with T2DM	Hybrid video + in-person, 12 months	Pre-intervention baseline	HbA1c +0·6% (*p* < 0·01); deterioration during pandemic
Debon (2020) ^S4^	Brazil	Non-randomised controlled pilot	39	Adults with hypertension	mHealth app, 3 months	Conventional monitoring	SBP intervention p < 0·01; control *p* = 0·67
Rodríguez-Montes (2025) ^S5^	Mexico	Pragmatic RCT	62	Adults with T2DM	mHealth app with AI-personalised messages	Standard care	HbA1c MD -0·20% (95% CI -0·65, 0·25)
Esandi (2024) ^S6^	Argentina	Pragmatic implementation study	3608 (*I* = 440)	Adults with hypertension, municipal	mHealth + community pharmacies, 24 weeks	Standard care	SBP MD -1·57 mmHg (*p* = 0·50); DBP MD -0·61 mmHg (p = 0·60)
Moreira (2025) ^S7^	Brazil	CONSORT-compliant RCT	147 (*I* = 73; C = 74)	Adults with T2DM transitioning to primary care	Nurse-led telephone support, 12 months	Standard transition	HbA1c between-group difference (95% CI -0·56%, 0·40%)
Cruz Gutiérrez (2023) ^S8^	Peru	Observational analytical	200	Adults with T2DM, primary care	Telephone follow-up, 6–28 months	Pre-intervention baseline	Proportion with HbA1c <8%: 74% → 67·5% (*p* = 0·05)
TELECONSULTA – Rodrigues (2024) ^S9^	Brazil	Phase 2 non-inferiority RCT	278 (*I* = 138; C = 140)	Adults with T2DM, public system	Video teleconsultation, 6 months	In-person consultation	HbA1c MD -0·5% (90% CI -0·9, 0·0) – non-inferior
Wilke-Franco (2022) ^S10^	Brazil	RCT during COVID-19	150 (*I* = 75; C = 75)	Adults with DM1 and DM2	Telemedicine + teleorientation, 16 weeks	Standard pandemic care	HbA1c: T2DM *p* = 0·58; T1DM p = 0·51 (NS)
Ramos-Zavala (2023) ^S11^	Mexico	Open-label RCT	178 (*I* = 94; C = 84)	Adults with uncontrolled hypertension	Home blood pressure telemonitoring, 12 weeks	Standard care	SBP MD -7·6 mmHg (*p* < 0·01); DBP -4·2 mmHg (*p* = 0·01)
Ciuffarella (2024) ^S12^	Guatemala	Cross-sectional study	68	Rural adults with HTN and/or T2DM	mHealth (calls + SMS), 5 months	Pre-intervention baseline	Capillary glycaemia p < 0·01; SBP/DBP NS
Calderón-Anyosa (2023) ^S13^	Peru	Two-arm RCT	38 (*I* = 19; C = 19)	Adults with hypertension, primary care	Adapted tensiometer + SMS, 4 weeks	Standard care	SBP MD −9·1 mmHg; DBP −6·0 mmHg; neither difference statistically significant
Spósito (2023) ^S14^	Uruguay	Prospective pre-post study	57	Adults with hypertension	Real-time blood pressure telemonitoring, 7 days	Pre-intervention baseline	SBP -7·1 mmHg (*p* = 0·02); DBP -3·9 mmHg (p = 0·06)
Aguiar (2018) ^S15^	Brazil	RCT	73 (*I* = 36; C = 37)	Adults with uncontrolled T2DM	Pharmacist-physician collaborative, 12 months	Standard care	HbA1c MD -0·63% (*p* = 0·01); LDL −10·2 mg/dL (p = 0·04) in DLP subset
Gómez (2022) ^S16^	Colombia	RCT	81 (*I* = 39; C = 42)	Adults with T2DM transitioning from inpatient	mHealth (ClouDi platform), 3 months	Standard care	HbA1c MD -2·0% (95% CI -3·39, −0·68)
Anzaldo-Campos - Dulce Wireless (2016) ^S17^	Mexico	Open-label 3-arm RCT	301	Adults with poorly controlled T2DM (HbA1c ≥8%)	Project Dulce + mobile, 10 months	Standard care	HbA1c MD -1·72% (*p* < 0·01) vs control
Lange (2010) ^S18^	Chile	Quasi-experimental longitudinal	432 (*I* = 206; C = 226)	Adults with T2DM, primary care	ATAS tele-care model, 15 months	Standard primary care	HbA1c +0·2% vs +1·4% (*p* < 0·01)
Díaz-Rueda (2018) ^S19^	Colombia	RCT	130 (*I* = 62; C = 68)	Adults with hypertension	Telephone + SMS, 2 months	Standard care	SBP/DBP NS (DBP *p* = 0·41); self-care 15% → 76%
Piette (2012) ^S20^	Honduras/Mexico	RCT	200 (181 analysed)	Adults with hypertension, rural	Cloud-based mobile + IVR, 6 weeks	Usual care	SBP MD -4·2 mmHg (95% CI -9·1, 0·7)
Kim (2020) ^S21^	Peru	Prospective pre-post	1271	Adults with hypertension, primary care	mHealth-supported programme, 24 months	Pre-intervention baseline	SBP 135·9 → 125·1; DBP 74·9 → 71·3 (all *p* < 0·01)
Khan (2024) ^S22^	Trinidad and Tobago	Retrospective pre-post observational	199	Adults with T2DM, HTN, and/or CHD	Telephone consultations during COVID-19, 7 months	Pre-pandemic in-person care	SBP *p* = 0·24; DBP p = 0·23; RBS p = 0·36 (all NS)

T2DM = type 2 diabetes mellitus. HTN = hypertension. CHD = coronary heart disease. BP = blood pressure. SBP = systolic blood pressure. DBP = diastolic blood pressure. IVR = interactive voice response. mHealth = mobile health. RBS = random blood sugar. NS = not significant. Superscript references refer to the Supplementary References list.

Among the 11 randomised trials, ten were judged to have some concerns and one high risk of bias, mainly relating to the randomisation process and missing outcome data; measurement of outcomes and selective reporting were generally low risk. Detailed risk-of-bias assessments are provided in Supplementary S5 File. Among non-randomised studies, five were rated serious and six moderate risk of bias, mainly from confounding and selection bias, with reporting bias generally low risk (Supplementary S5 File).

Of the 22 included studies, clinically comparable randomised trials contributed to the quantitative syntheses: seven trials (935 participants) for HbA1c, four (451 participants) for systolic blood pressure, and three (280 participants) for diastolic blood pressure. Remaining outcomes were synthesised narratively because of insufficient comparable randomised evidence or heterogeneity in intervention design and outcome reporting. HbA1c trials evaluated mobile applications, telephone follow-up, video consultations, telemonitoring, and multicomponent interventions, generally versus usual, standard, or active in-person care over approximately 3–12 months. Blood-pressure trials evaluated telemonitoring, mobile-health support, telephone management, and clinician-supported remote care, predominantly versus usual or standard care over approximately 1–12 months.

Methodological approaches varied among the trials contributing to the meta-analyses. Intention-to-treat analyses were used in some, but not all, contributing trials, and both adjusted and unadjusted effect estimates were reported. Attrition and approaches to missing data also varied, with some trials using modified intention-to-treat or imputation approaches and others analysing available cases. Whether cardiometabolic outcomes were explicitly designated as primary or secondary endpoints was not consistently reported across studies.

The HbA1c meta-analysis (seven trials; 935 participants) showed a pooled mean difference of −0·62% (95% CI -1·02, −0·22; I^2^ = 66%, τ^2^ = 0·18). The 95% prediction interval (−1·77%, 0·56%) crossed the null, indicating that although the pooled estimate favoured telemedicine, the effect may vary across clinical settings and intervention types, and benefit cannot be assumed in all settings. Certainty of evidence was low (Supplementary S6 File). Sensitivity analyses did not alter the direction or significance of the pooled estimate (Supplementary S7 File). One eligible trial (Díaz-Rueda, 2018 [S19]) was excluded from pooling for lack of clinical comparability. The systolic blood pressure meta-analysis (four trials; 451 participants) showed a pooled mean difference of −6·60 mmHg (95% CI -9·49, −3·71; I^2^ = 0%), with high certainty of evidence (Fig. 3; Supplementary S6 File). The diastolic blood pressure meta-analysis (three trials; 280 participants) showed a pooled mean difference of −4·25 mmHg (95% CI -6·53, −1·98; I^2^ = 0%), with moderate certainty of evidence (Supplementary S6 File).

The absence of detectable statistical heterogeneity (I^2^ = 0%) should be interpreted cautiously given the small number of contributing trials and should not be considered evidence of clinical homogeneity. Sensitivity analyses using alternative τ^2^ estimators and leave-one-out methods did not materially change these estimates (Supplementary S7 File).

At the trial level, the largest HbA1c reduction was reported by Gomez and colleagues (2022) [S16], among patients transitioning from inpatient to outpatient care with baseline HbA1c near 12% (−2·0%, 95% CI -3·39, −0·68); Anzaldo-Campos and colleagues (2016) [S17] reported a − 1·72% reduction with the technology-enhanced Project Dulce programme among patients with HbA1c 8% or higher (*p* < 0·01), and Aguiar and colleagues (2018) [S15] reported a − 0·63% reduction (95% CI -1·09, −0·17) after pharmacist-physician collaboration combined with telephone follow-up. Lower-intensity app-based messaging, telephone follow-up, and pandemic-era interventions generally showed modest or null between-group differences [S5,S7,S10]; the TELECONSULTA trial demonstrated non-inferiority of video consultations versus face-to-face care in the Brazilian public health system (mean difference − 0·5%, 90% CI -0·9, 0·0) [S9]. Non-randomised studies provided concordant but methodologically weaker evidence: Lange and colleagues (2010) [S18] reported a − 1·2% between-group difference in a Chilean quasi-experimental study of 432 patients in primary care (*p* < 0·01), Piette and colleagues (2011) [S2] reported a within-group reduction of 1·1% among adherent users of an interactive voice response programme in Honduras, and Cruz Gutierrez and colleagues (2023) [S8] reported a decline in the proportion of Peruvian patients achieving HbA1c below 8% over up to 28 months of follow-up (74% to 67·5%; *p* = 0·05).

For blood pressure, Ramos-Zavala and colleagues (2023) [S11] reported reductions of −7·6 mmHg (systolic) and − 4·2 mmHg (diastolic) after 12 weeks of app-supported home telemonitoring (both *p* < 0·01), and Calderón-Anyosa and colleagues (2023) [S13] reported reductions of −9·1 mmHg and − 6·0 mmHg with SMS-linked home monitoring, although neither between-group difference reached statistical significance. In the hypertensive subgroup of Aguiar and colleagues [S15], systolic pressure fell by 12·5 mmHg versus 4·4 mmHg with standard care (*p* < 0·01), and diastolic pressure fell by 6·3 versus 3·2 mmHg (*p* = 0·01); Piette and colleagues (2012) [S20] reported a smaller systolic reduction that did not reach statistical significance, and Diaz-Rueda and colleagues (2018) [S19] found insufficient evidence of a blood-pressure difference despite increased self-care capacity. Among non-randomised studies, Kim and colleagues (2020) [S21] reported pre-post reductions of −10·8 mmHg (systolic) and − 3·6 mmHg (diastolic) over 24 months (both *p* < 0·01), and Spósito and colleagues (2023) [S14] reported short-term reductions of −7·1 mmHg and − 3·9 mmHg after real-time telemonitoring, whereas Esandi and colleagues (2024) [S6], the largest implementation study, found small, non-significant differences (systolic −1·57 mmHg; diastolic −0·61 mmHg), and Khan and colleagues (2024) [S22] reported no significant changes in blood pressure or random blood glucose before versus during pandemic-era telemedicine (all *p* > 0·20).

Lipid outcomes were infrequently and inconsistently reported; Aguiar and colleagues [S15] reported a between-group LDL-cholesterol reduction (−10·2 mg/dL, 95% CI -20·1, −0·3) in a dyslipidaemic subgroup, while other studies reported imprecise or non-significant within-group changes [S3,S10,S17,S21]. No study reported between-group HDL-cholesterol, triglycerides, or total cholesterol, and none was designed specifically to evaluate dyslipidaemia. Consequently, the available evidence was insufficient to establish either benefit or absence of effect for most lipid outcomes.

Secondary outcomes were reported unevenly (Supplementary S4 File). Aguiar and colleagues [S15] was the only study to report a validated between-group medication-adherence measure, showing greater improvement in Morisky Green 4 scores with the intervention (*p* < 0·01). Patient satisfaction generally exceeded 90% [S2,S11], except in the TELECONSULTA trial, where satisfaction was slightly lower with telemedicine but remained high [S9]; engagement was variable, with completion rates above 70% in several trials but 77% attrition in the largest implementation study [S6]. Only two studies assessed health-care utilisation: Gomez and colleagues [S16] reported lower 30-day readmission with the intervention (2·5% vs 15·2%), whereas Moreira and colleagues (2025) [S7] found insufficient evidence of a difference in emergency-department visits; no study assessed mortality, cardiovascular events, or intensive-care admissions, and none performed formal cost-effectiveness analyses.

Reporting of equity-related variables was limited and inconsistent: most studies reported participant sex and age, but few reported ethnicity, educational attainment, income, employment status, or other social determinants of health, and several interventions required smartphone ownership, internet connectivity, or basic digital literacy as eligibility criteria, potentially excluding socially vulnerable populations [S4–S6,S11,S13]. Rural populations were specifically represented in only a minority of studies [S1,S2,S12,S20], and no study performed stratified analyses by sex, gender, socioeconomic status, ethnicity, or educational attainment. Telemedicine interventions were more frequently implemented in urban or semi-urban settings linked to structured primary-care programmes or academic institutions; although mobile-phone coverage has expanded substantially across LAC, disparities in internet access, smartphone availability, and digital literacy remain strongly associated with income, educational level, age, and rural residence (*Economic Commission for Latin America and the Caribbean (ECLAC). Technology, Digitalization and Equality: Institutional Frameworks for Inclusive Digital Development*, 2021; World Bank; United Nations Development Programme, 2022; International Telecommunication Union, 2023), so the available evidence likely overrepresents populations with greater digital connectivity and health-care access, and no included study explicitly evaluated whether telemedicine reduced or widened pre-existing health-care inequities.

Certainty of evidence was formally rated with GRADE for the pooled HbA1c and blood-pressure outcomes. HbA1c was rated low certainty because of serious inconsistency and imprecision; systolic blood pressure was rated high certainty, with no downgrading in any GRADE domain; and diastolic blood pressure was rated moderate certainty because of serious imprecision (Supplementary S6 File). Lipid outcomes and adherence/self-care outcomes were not assigned a single GRADE certainty rating because they were synthesised narratively and did not constitute sufficiently homogeneous bodies of evidence. Because each pooled analysis included fewer than ten trials, funnel plots were inspected descriptively only, and no formal test for small-study effects or publication bias was done.

## 4 Discussion

In this systematic review of 22 studies from ten LAC countries, the pooled estimates favoured telemedicine, with reductions of −0·62% in HbA1c, −6·60 mmHg in systolic blood pressure, and − 4·25 mmHg in diastolic blood pressure (Fig. 2, Fig. 3; Supplementary Fig. S1). These effect sizes may be clinically relevant: the observed HbA1c reduction is of a magnitude that could contribute meaningfully to glycaemic control, while the blood-pressure reductions are potentially important given the established relationship between blood-pressure lowering and cardiovascular risk reduction (American diabetes association professional practice Committee for Diabetes, 2026; McEvoy et al., 2024).

**Fig. 2:**
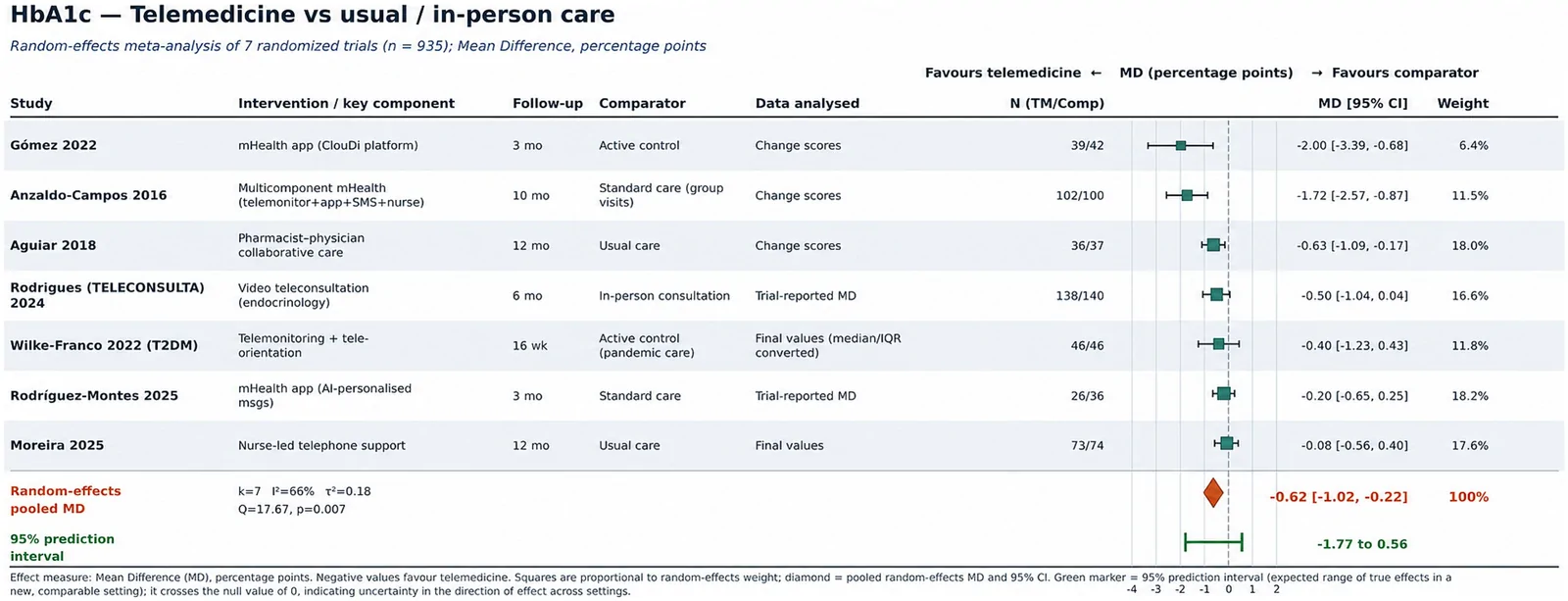
Random-effects meta-analysis of glycated haemoglobin among adults with type 2 diabetes mellitus in randomised trials comparing telemedicine with usual, standard, active, or in-person care in Latin America and the Caribbean, with follow-up of approximately 3–12 months; searches were completed on 17 December 2025. Random-effects meta-analysis of HbA1c. Squares are proportional to study weight; the diamond represents the pooled estimate.

**Fig. 3:**
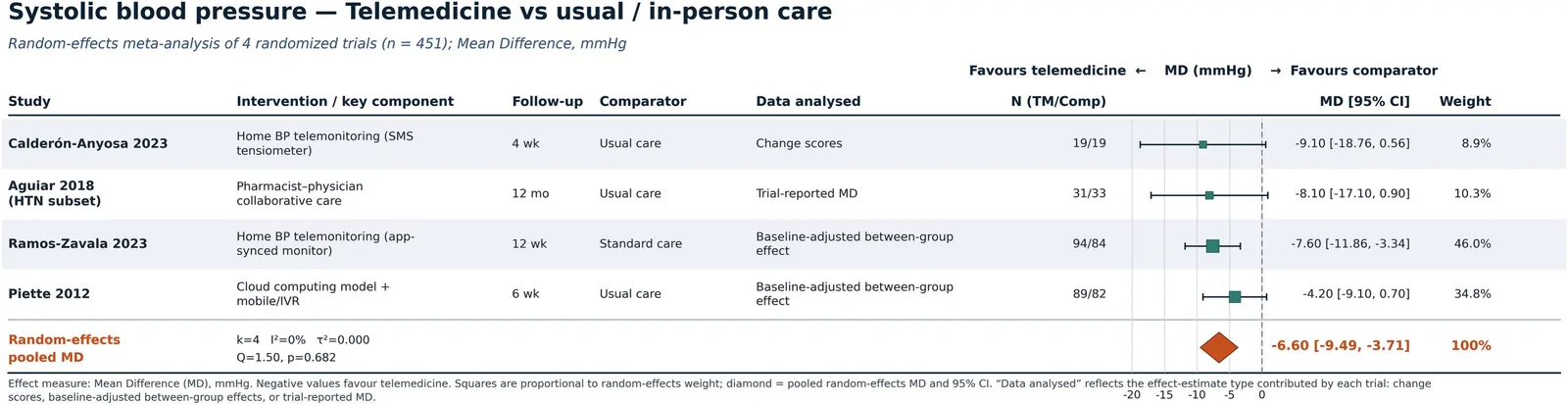
Random-effects meta-analysis of systolic blood pressure among adults with hypertension in randomised trials comparing telemedicine with usual or standard care in Latin America and the Caribbean, with follow-up of approximately 1–12 months; searches were completed on 17 December 2025. Random-effects meta-analysis of systolic blood pressure among randomised trials evaluating telemedicine interventions for hypertension in Latin America and the Caribbean.

Blood-pressure estimates showed less statistical heterogeneity, whereas HbA1c estimates showed substantial heterogeneity and a 95% prediction interval crossing the null (−1·77%, 0·56%). Thus, although the pooled estimate favoured telemedicine, a benefit may not be observed in all future comparable settings, and the magnitude and direction of the effect may vary according to baseline disease severity, intervention intensity, follow-up duration, and clinical context. Similarly, the absence of detectable statistical heterogeneity in the blood-pressure meta-analyses should not be interpreted as clinical homogeneity, given the small number of contributing trials and differences in intervention content, participant characteristics, and healthcare settings.

This review provides the first quantitative synthesis of telemedicine interventions for cardiometabolic disease in LAC. Telemedicine is not a uniform intervention, and differences in content, intensity, clinician involvement, and healthcare context may contribute to variation in treatment effects. However, these observations are exploratory and hypothesis-generating because they were not formally evaluated through subgroup analysis or meta-regression.

Baseline disease severity probably contributed to between-study variation: larger HbA1c reductions occurred among patients with poorer baseline control [S16,S17], whereas better-controlled populations showed smaller or null effects [S4,S6], and one observational study reported a decline in the proportion achieving HbA1c below 8% during follow-up, potentially reflecting pandemic-related disruption to care rather than a direct intervention effect [S8]. A similar pattern was seen in hypertension studies, where interventions targeting uncontrolled hypertension produced larger blood-pressure reductions than pragmatic implementation studies, and regression to the mean may have inflated some estimates in trials without concurrent comparators or with markedly elevated baseline values [S11,S13,S20]. The observed benefits cannot be attributed to telemedicine alone, because several interventions also intensified clinical care through medication titration, interpretation of remotely collected data, personalised feedback, multidisciplinary management, or more frequent clinician follow-up [S5,S7,S15–S17]. These additional components may have contributed to the observed effects independently of the mode of care delivery, particularly in open-label trials without attention-matched comparators (Kebede and Pischke, 2019; Omboni et al., 2020). Differences in comparator conditions, ranging from structured outpatient follow-up to delayed or fragmented routine care, together with variability in follow-up duration, outcome measurement, and attrition, probably also contributed to heterogeneity, and non-randomised or single-arm studies generally reported larger within-group improvements than randomised trials, underscoring the importance of concurrent controls.

The pooled estimates were broadly consistent with previous telemedicine evidence reporting HbA1c reductions of approximately 0·3–0·6% and systolic blood pressure reductions of 3–6 mmHg (Kebede and Pischke, 2019; Omboni et al., 2020). Differences in effect magnitude may reflect intervention intensity, clinician involvement, follow-up duration, eligibility criteria, baseline disease severity, and comparator care. Several studies in this review enrolled participants with uncontrolled disease and evaluated interventions incorporating remote monitoring or active treatment adjustment [S11,S13,S15–S17], which may partly explain the larger reductions observed in some trials, whereas shorter follow-up, pragmatic eligibility criteria, and pandemic-related changes in usual care may have attenuated effects in others [S6,S7,S10]. Healthcare context may also contribute to differences from previous evidence: in fragmented LAC health systems, telemedicine may expand access and enable treatment intensification amid workforce shortages and barriers to care (Atun et al., 2015; Cotlear et al., 2015), whereas in high-income settings it more often provides an alternative modality within established chronic-disease services (Kebede and Pischke, 2019; Omboni et al., 2020). Standard care also varies across and within LAC health systems (Atun et al., 2015; Cotlear et al., 2015), and the rapid expansion of telemedicine during COVID-19 altered intervention delivery and comparator conditions (World Bank; United Nations Development Programme, 2022; International Telecommunication Union, 2023; Atun et al., 2015; Cotlear et al., 2015). These differences in study characteristics and healthcare context limit direct comparison with previous reviews and the extrapolation of evidence from high-income settings to LAC.

Reporting of equity-related variables was limited, and most studies enrolled participants with access to mobile phones, connectivity, or basic digital literacy [S4–S6,S11,S13], probably underrepresenting rural, Indigenous, Afro-descendant, and lower-income populations, among whom digital disparities persist (*Economic Commission for Latin America and the Caribbean (ECLAC). Technology, Digitalization and Equality: Institutional Frameworks for Inclusive Digital Development*, 2021; World Bank; United Nations Development Programme, 2022; International Telecommunication Union, 2023). No study assessed whether telemedicine narrowed or widened pre-existing healthcare inequities. Although selected cardiometabolic outcomes showed potential clinical benefit, the evidence does not establish feasibility for large-scale implementation. Evidence on scalability, sustainability, cost-effectiveness, equitable access, and integration into routine healthcare was limited, as few studies systematically evaluated implementation outcomes [S4–S6,S11,S13] (*Economic Commission for Latin America and the Caribbean (ECLAC). Technology, Digitalization and Equality: Institutional Frameworks for Inclusive Digital Development*, 2021; World Bank; United Nations Development Programme, 2022; International Telecommunication Union, 2023). Future implementation research should assess these outcomes and relevant social determinants, including socioeconomic status, education, residence, and ethnicity (O'Neill et al., 2014; Karran et al., 2023; *Economic Commission for Latin America and the Caribbean (ECLAC). Technology, Digitalization and Equality: Institutional Frameworks for Inclusive Digital Development*, 2021; World Bank; United Nations Development Programme, 2022; International Telecommunication Union, 2023).

This review's strengths include a prospectively registered protocol, comprehensive searches of international and regional databases and grey literature, inclusion of English, Spanish, and Portuguese-language studies, duplicate screening and extraction, and formal risk-of-bias and GRADE assessments. Limitations include the small number of studies contributing to each meta-analysis, which precluded prespecified subgroup analyses and meta-regression and limited formal investigation of heterogeneity, particularly for HbA1c (I^2^ = 66%). Clinical and methodological variability in intervention content, intensity, populations, comparators, and follow-up further restricted quantitative synthesis. Risk of bias was also a concern, with some concerns or high risk among randomised trials and moderate-to-serious risk among non-randomised studies. Finally, the evidence relied predominantly on intermediate cardiometabolic outcomes, with limited evidence on long-term effectiveness, cardiovascular events, healthcare utilisation, cost-effectiveness, and implementation. Several included studies were conducted during the COVID-19 pandemic, when health-care delivery and comparator conditions changed substantially. The rapid expansion of remote care may have attenuated between-group contrasts, limiting generalisability to post-pandemic settings (Pan American Health Organization, 2020; Atun et al., 2015; Cotlear et al., 2015). Generalisability is also limited by uneven geographical and healthcare-setting representation, with Brazil and Mexico accounting for nearly half of included studies (Table 1) and relatively few studies representing rural populations. Applicability to other LAC countries, rural populations, and settings with limited connectivity, digital infrastructure, or access to digital technologies therefore remains uncertain. Some regional reports may have been missed despite extensive grey-literature searches, five reports could not be retrieved, and formal assessment of small-study effects and publication bias was not feasible given the limited number of comparable studies; pooling was also restricted to randomised trials because non-randomised and pre-post studies showed major methodological heterogeneity and high susceptibility to confounding.

Future research in LAC should prioritise pragmatic, multicountry randomised trials of standardised telemonitoring and treatment-adjustment strategies embedded in public primary-care systems, with robust assessment of long-term cardiovascular outcomes, health-care utilisation, and cost-effectiveness (Omboni et al., 2020; Ogungbe et al., 2025), and should incorporate implementation-science frameworks to evaluate scalability, sustainability, and equity, particularly among rural populations and groups with limited digital access (O'Neill et al., 2014; Karran et al., 2023; *Economic Commission for Latin America and the Caribbean (ECLAC). Technology, Digitalization and Equality: Institutional Frameworks for Inclusive Digital Development*, 2021; World Bank; United Nations Development Programme, 2022; International Telecommunication Union, 2023). Given that most included studies had follow-up of six months or less, future research should determine whether improvements are sustained beyond one year and translate into reductions in major cardiovascular events and health-system burden (Kebede and Pischke, 2019; Omboni et al., 2020).

## 5 Conclusions

Selected telemedicine interventions may improve intermediate cardiometabolic outcomes, particularly HbA1c and blood pressure, in specific LAC settings. However, these findings are based on a limited number of heterogeneous studies, with variable certainty of evidence and substantial heterogeneity in the HbA1c analysis. Generalisability across LAC remains uncertain, particularly for countries, rural populations, and settings with limited digital resources underrepresented in the evidence. Further research is needed to establish long-term effectiveness and to evaluate the feasibility, scalability, sustainability, cost-effectiveness, equity, and integration of telemedicine into routine care across diverse healthcare settings.

## Data sharing

The data underlying this study are derived from published studies included in this systematic review and meta-analysis. All extracted data supporting the findings are available within the article and its supplementary materials.

## CRediT authorship contribution statement

**Kevin Saldarriaga-Bedoya:** Writing – review & editing, Writing – original draft, Visualization, Validation, Software, Methodology, Investigation, Funding acquisition, Formal analysis, Data curation, Conceptualization. **Sergio César Ruiz-Jiménez:** Writing – review & editing, Writing – original draft, Validation, Supervision, Methodology, Investigation, Formal analysis, Data curation, Conceptualization. **Santiago José Sánchez-Rodríguez:** Writing – review & editing, Writing – original draft, Visualization, Validation, Methodology, Investigation, Data curation, Conceptualization. **Gladys Rocío Ariza-Sosa:** Writing – review & editing, Writing – original draft, Supervision, Methodology, Formal analysis, Data curation, Conceptualization. **Sjoukje van der Werf:** Writing – review & editing, Writing – original draft, Validation, Supervision, Methodology, Investigation, Data curation, Conceptualization. **Erik Buskens:** Writing – review & editing, Writing – original draft, Validation, Supervision, Investigation, Data curation, Conceptualization. **Maarten Lahr:** Writing – review & editing, Writing – original draft, Supervision, Software, Methodology, Investigation, Formal analysis, Conceptualization.

## Ethics statement

Ethical approval and informed consent were not required for this systematic review because all data were derived from previously published studies and publicly available sources, with no collection or analysis of individual-level identifiable participant data.

## Ethics declaration

### Informed consent and patient details

Written informed consent to take part in the study and to publish the article has been obtained from all participants or their legal representatives. The privacy rights of participants have been observed.

### Studies in human

This study was performed in compliance with relevant laws, regulatory frameworks and guidelines where the research took place.

This study was approved by the Universidad de antioquia. (Approval No. acta 177 no 109).

## Funding source

This research did not receive any specific grant from funding agencies in the public, commercial, or not-for-profit sectors.

## Declaration of competing interest

The authors declare the following financial interests/personal relationships which may be considered as potential competing interests: KEVIN SALDARRIAGA BEDOYA reports financial support was provided by University Medical Centre Groningen. If there are other authors, they declare that they have no known competing financial interests or personal relationships that could have appeared to influence the work reported in this paper.

## Data Availability

Data were derived from published studies. All extracted data supporting the findings are available within the article and its supplementary materials.

## Appendix A Supplementary data

Supplementary material 1: S1 File. Full database-by-database search strategies for PubMed/MEDLINE, Embase, Web of Science, Scopus, LILACS, Cochrane CENTRAL, and BVS, with execution dates and record yields.

Supplementary material 2: S2 File. Completed 27-item PRISMA 2020 checklist with manuscript location of each item.

Supplementary material 3: S3 File. Full-text exclusions: list of 93 exclusions with title, year, author, journal, DOI, and primary reason; separate sheet listing the five reports not retrieved.

Supplementary material 4: S4 File. Full data-extraction template (156 variables) for the 22 included studies.

Supplementary material 5: S5 File. Domain-level risk-of-bias assessments (RoB 2 and ROBINS-I) with justifications, including the risk-of-bias summary bar chart (RoB 2 domains), the RoB 2 traffic-light plot, the ROBINS-I traffic-light plot, and the overall risk-of-bias judgement per included study.

Supplementary material 6: S6 File. Additional meta-analysis results and GRADE Summary of Findings tables for HbA1c, systolic and diastolic blood pressure, lipid outcomes, and adherence/self-care outcomes.

Supplementary material 7: S7 File. Sensitivity analyses: leave-one-out analyses and restricted-maximum-likelihood tau^2^ estimator results for HbA1c, systolic blood pressure, and diastolic blood pressure.

Supplementary material 8: S8 File. Within-group analyses of non-randomised studies.
