# Supplementary material for: Telemedicine for cardiometabolic disease control in Latin America and the Caribbean: A systematic review and meta-analysis
Source: Prev Med Rep. 2026 Sep 23;71:103633. doi: 10.1016/j.pmedr.2026.103633 (PMC13634337; doi:10.1016/j.pmedr.2026.103633)
Supplement: Supplementary file 1 [file mmc1.docx]

**Final search strategies**

**Search results 17 December 2025**

A. PubMed – 1490
B. Embase – 1773
C. Web of Science + SciELO – 1616
D. Cochrane CENTRAL – 879
E. Scopus – 1678
F. LILACS – 1173
G. BVS – 39
H. PAHO/WHO IRIS – 25
I. RedALyC – 276
J. Latindex – 0 (journals only, not articles)
K. Repositories (LA Referencia, SNRD, CONACYT, ALICIA, etc.) – 42
L. CLACSO Repositorio – 0

**PubMed search strategy**

("Hypertension"[Mesh] OR "Prehypertension"[Mesh] OR "Diabetes Mellitus"[Mesh] OR "Dyslipidemias"[Mesh] OR "Glycated Hemoglobin"[Mesh] OR "Blood Pressure"[Mesh:NoExp] OR "Cholesterol"[Mesh] OR "Blood Glucose"[Mesh] OR "Glycemic Control"[Mesh] OR "Blood Pressure Monitoring, Ambulatory"[Mesh] OR "hypertension"[tiab] OR "prehypertension*"[tiab] OR "diabet*"[tiab] OR "dyslipidem*"[tiab] OR "hyperlipid*"[tiab] OR "elevated cholesterol"[tiab] OR "high cholesterol"[tiab] OR "hypercholesterol*"[tiab] OR "cardiometabolic*"[ti] OR "metabolic"[ti] OR "glycemic control"[tiab] OR "glycaemic control"[tiab] OR "blood pressure"[tiab] OR "lipid profile*"[tiab] OR "hba1c"[tiab] OR "blood glucose"[tiab])

AND   
("Telecommunications"[Mesh:NoExp] OR "Telemedicine"[Mesh] OR "Telemetry"[Mesh] OR "Videoconferencing"[Mesh] OR "Telephone"[Mesh:NoExp] OR "Cell Phone"[Mesh] OR "Mobile Applications"[Mesh] OR ("tele*"[ti] NOT ("television"[ti] OR "telephone"[ti])) OR "teleconsult*"[tiab] OR "telehealth"[tiab] OR "telemedicine"[tiab] OR "telemed*"[tiab] OR "e consult*"[tiab] OR "virtual consult*"[tiab] OR "digital consult*"[tiab] OR "online consult*"[tiab] OR "internet consult*"[tiab] OR "remote consult*"[tiab] OR "video consult*"[tiab] OR "digital intervention*"[tiab] OR "mhealth"[tiab] OR "m health"[tiab] OR "ehealth"[tiab] OR "e health"[tiab] OR "e consult*"[tiab] OR "e care"[tiab] OR "e management"[tiab] OR "technology enhanced"[tiab] OR "text messag*"[tiab] OR  
"remote consult"[tiab:~3] OR "remote consultation"[tiab:~3] OR "remote consultations"[tiab:~3] OR "remote appointment"[tiab:~3] OR "remote appointments"[tiab:~3] OR "remote intervention"[tiab:~3] OR "remote interventions"[tiab:~3] OR "remote monitoring"[tiab:~3] OR "remote care"[tiab:~3] OR "remote management"[tiab:~3] OR "remote glycemic control"[tiab:~3] OR "online consult"[tiab:~3] OR "online consultation"[tiab:~3] OR "online consultations"[tiab:~3] OR "online appointment"[tiab:~3] OR "online appointments"[tiab:~3] OR "online intervention"[tiab:~3] OR "online interventions"[tiab:~3] OR "online monitoring"[tiab:~3] OR "online care"[tiab:~3] OR "online management"[tiab:~3] OR "online glycemic control"[tiab:~3] OR "internet consult"[tiab:~3] OR "internet consultation"[tiab:~3] OR "internet consultations"[tiab:~3] OR "internet appointment"[tiab:~3] OR "internet appointments"[tiab:~3] OR "internet intervention"[tiab:~3] OR "internet interventions"[tiab:~3] OR "internet monitoring"[tiab:~3] OR "internet care"[tiab:~3] OR "internet management"[tiab:~3] OR "internet glycemic control"[tiab:~3] OR "digi consult"[tiab:~3] OR "digi consultation"[tiab:~3] OR "digi consultations"[tiab:~3] OR "digi appointment"[tiab:~3] OR "digi appointments"[tiab:~3] OR "digi intervention"[tiab:~3] OR "digi interventions"[tiab:~3] OR "digi monitoring"[tiab:~3] OR "digi care"[tiab:~3] OR "digi management"[tiab:~3] OR "digi glycemic control"[tiab:~3] OR "digital consult"[tiab:~3] OR "digital consultation"[tiab:~3] OR "digital consultations"[tiab:~3] OR "digital appointment"[tiab:~3] OR "digital appointments"[tiab:~3] OR "digital intervention"[tiab:~3] OR "digital interventions"[tiab:~3] OR "digital monitoring"[tiab:~3] OR "digital care"[tiab:~3] OR "digital management"[tiab:~3] OR "digital glycemic control"[tiab:~3] OR "tele consult"[tiab:~3] OR "tele consultation"[tiab:~3] OR "tele consultations"[tiab:~3] OR "tele appointment"[tiab:~3] OR "tele appointments"[tiab:~3] OR "tele intervention"[tiab:~3] OR "tele interventions"[tiab:~3] OR "tele monitoring"[tiab:~3] OR "tele care"[tiab:~3] OR "tele management"[tiab:~3] OR "tele glycemic control"[tiab:~3] OR "virtual consult"[tiab:~3] OR "virtual consultation"[tiab:~3] OR "virtual consultations"[tiab:~3] OR "virtual appointment"[tiab:~3] OR "virtual appointments"[tiab:~3] OR "virtual intervention"[tiab:~3] OR "virtual interventions"[tiab:~3] OR "virtual monitoring"[tiab:~3] OR "virtual care"[tiab:~3] OR "virtual management"[tiab:~3] OR "virtual glycemic control"[tiab:~3] OR "distance consult"[tiab:~3] OR "distance consultation"[tiab:~3] OR "distance consultations"[tiab:~3] OR "distance appointment"[tiab:~3] OR "distance appointments"[tiab:~3] OR "distance intervention"[tiab:~3] OR "distance interventions"[tiab:~3] OR "distance monitoring"[tiab:~3] OR "distance care"[tiab:~3] OR "distance management"[tiab:~3] OR "distance glycemic control"[tiab:~3] OR "video consult"[tiab:~3] OR "video consultation"[tiab:~3] OR "video consultations"[tiab:~3] OR "video appointment"[tiab:~3] OR "video appointments"[tiab:~3] OR "video intervention"[tiab:~3] OR "video interventions"[tiab:~3] OR "video monitoring"[tiab:~3] OR "video care"[tiab:~3] OR "video management"[tiab:~3] OR "video glycemic control"[tiab:~3] OR (("online based"[tiab] OR "webbased"[tiab] OR "web based"[tiab] OR "computer based"[tiab] OR "internetbased"[tiab] OR "video based"[tiab] OR "virtual"[tiab] OR "remote"[ti] OR "technology based"[ti] OR "mobile based"[ti] OR "app based"[ti] OR "wearab*"[ti]) AND ("intervention*"[ti] OR "program*"[ti] OR "care"[ti] OR "trial"[ti] OR "consult*"[ti] OR "support"[ti] OR "service*"[ti] OR "management"[ti] OR "monitoring"[ti])) OR "ambulatory blood pressure monitoring"[tiab:~3] OR "home blood pressure monitoring"[tiab:~3] OR "mobile technology blood pressure monitoring"[tiab:~3] OR "mobile application"[ti:~3] OR "mobile app*"[ti])

AND

( "Clinical Trial"[Publication Type] OR "Cohort Studies"[Mesh] OR "Program Evaluation"[Mesh] OR "Comparative Study" [Publication Type] OR "trial"[tiab] OR "trials"[tiab] OR "random*"[tiab] OR "controlled study"[tiab] OR "program*"[ti] OR "pilot study"[tiab] OR "intervention*"[ti] OR "cohort"[tiab] OR (("follow up"[tiab] OR "prospective"[tiab] OR "retrospective"[tiab]) AND "study"[tiab]))

AND

( "Latin America"[Mesh] OR "Hispanic or Latino"[Mesh] OR "South America"[Mesh] OR "Central America"[Mesh] OR "Caribbean Region"[Mesh] OR "low middle income countries"[tiab:~3] OR "lower middle income countries"[tiab:~3] OR "latin*"[tw] OR "hispanic*"[tw] OR "south americ*"[tw] OR "central americ*"[tw] OR (( "portuges*"[tw] OR "spanish"[tw]) AND "america*"[tiab]) OR "argentin*"[tw] OR "argentina"[ad] OR "bolivi*"[tw] OR "bolivia"[ad] OR "brazil*"[tw] OR "brasil*"[tw] OR "brazil"[ad] OR "brasil"[ad] OR "chil*"[tw] OR "chile"[ad] OR "colombi*"[tw] OR "colombia"[ad] OR "costa ric*"[tw] OR "costa rica"[ad] OR "cuba*"[tw] OR "cuba"[ad] OR "dominican republic"[tw] OR "dominican republic"[ad] OR "ecuador*"[tw] OR "ecuador"[ad] OR "el salvador"[tw] OR "el salvador"[ad] OR "guatemal*"[tw] OR "guatemala"[ad] OR "hondura*"[tw] OR "honduras"[ad] OR "mexic*"[tw] OR "mexico"[ad] OR "nicaragu*"[tw] OR "nicaragua"[ad] OR "panam*"[tw] OR "panama"[ad] OR "paraguay*"[tw] OR "paraguay"[ad] OR "peru*"[tw] OR "peru"[ad] OR "puerto ric*"[tw] OR "puerto rico"[ad] OR "uruguay*"[tw] OR "uruguay"[ad] OR "venezuel*"[tw] OR "venezuela"[ad] OR "caribbean"[tw] OR "caribbean"[ad] OR "aruba"[tw] OR "aruba"[ad] OR "curacao"[tw] OR "curacao"[ad] OR "sint maarten"[tw] OR "sint maarten"[ad] OR "saint martin"[tw] OR "saint martin"[ad] OR "bonaire"[tw] OR "bonaire"[ad] OR "sint eusta*"[tw] OR "sint eusta*"[ad] OR "saint eusta*"[tw] OR "saint eusta*"[ad] OR "saba"[tw] OR "saba"[ad] OR "antilles"[tw] OR "antilles"[ad] OR "caribbean"[tw] OR "caribbean"[ad] OR "antigua"[tw] OR "barbuda"[tw] OR "antigua"[ad] OR "barbuda"[ad] OR "bahamas"[tw] OR "bahamas"[ad] OR "barbados"[tw] OR "barbados"[ad] OR "virgin islands"[tw] OR "virgin islands"[ad] OR "dominica"[tw] OR "dominica"[ad] OR "grenada"[tw] OR "grenada"[ad] OR "guadeloupe"[tw] OR "guadeloupe"[ad] OR "haiti"[tw] OR "haiti"[ad] OR "jamaica"[tw] OR "jamaica"[ad] OR "martinique"[tw] OR "martinique"[ad] OR "saint kitts"[tw] OR "saint kitts"[ad] OR "saint lucia"[tw] OR "saint lucia"[ad] OR "saint vincent"[tw] OR "saint vincent"[ad] OR "trinidad"[tw] OR "trinidad"[ad] OR "tobago"[tw] OR "tobago"[ad] )

NOT

( (("Child"[Mesh] OR "Adolescent"[Mesh]) NOT "Adult"[Mesh]) OR (( "child*"[ti] OR "pediat*"[ti] OR "paediat*"[ti]) NOT "adult*"[ti]) )  

Embase (embase.com)

('hypertension'/de OR 'prehypertension'/exp OR 'diabetes mellitus'/exp OR 'dyslipidemia'/exp OR 'glycated hemoglobin'/exp OR 'blood pressure'/de OR 'disorders of cholesterol metabolism'/exp OR 'glucose blood level'/exp OR 'glycemic control'/exp OR 'blood pressure monitoring'/exp OR 'hypertension':ti,ab,kw OR 'prehypertension*':ti,ab,kw OR 'diabet*':ti,ab,kw OR 'dyslipidem*':ti,ab,kw OR 'hyperlipid*':ti,ab,kw OR 'elevated cholesterol':ti,ab,kw OR 'high cholesterol':ti,ab,kw OR 'glycemic control':ti,ab,kw OR 'glycaemic control':ti,ab,kw OR 'blood pressure':ti,ab,kw OR 'lipid profile*':ti,ab,kw OR 'hba1c':ti,ab,kw OR 'blood glucose':ti,ab,kw OR 'cardiometabolic*':ti OR 'metabolic':ti)

AND

('telecommunication'/de OR 'telemedicine'/exp OR 'telehealth'/exp OR 'telemetry'/exp OR 'videoconferencing'/exp OR 'telephone'/de OR 'mobile phone'/exp OR 'mobile application'/exp
OR ('tele*' NOT ('television' OR 'telephone')):ti
OR
('teleconsult*' OR 'telehealth' OR 'telemedicine' OR 'telemed*' OR 'e consult*' OR 'virtual consult*' OR 'digital consult*' OR 'online consult*' OR 'internet consult*' OR 'remote consult*' OR 'video consult*' OR 'digital intervention*' OR 'mhealth' OR 'm health' OR 'ehealth' OR 'e health' OR 'e consult*' OR 'e care' OR 'e management' OR 'technology enhanced' OR 'text messag*'):ti,ab,kw
OR
(('remote' OR 'online' OR 'internet' OR 'digi*' OR 'tele' OR 'virtual' OR 'distance' OR 'video') NEAR/4 ('consult*' OR 'appointment*' OR 'intervention*' OR 'monitoring' OR 'care' OR 'management')):ti,ab,kw
OR
(('remote' OR 'online' OR 'internet' OR 'digi*' OR 'tele' OR 'virtual' OR 'distance' OR 'video') NEAR/4 'glycemic control'):ti,ab,kw
OR
(('online based' OR 'webbased' OR 'web based' OR 'computer based' OR 'internetbased' OR 'video based'):ti,ab,kw *AND* ('intervention*' OR 'program*' OR 'care' OR 'trial' OR 'consult*' OR 'support' OR 'service*' OR 'management' OR 'monitoring'):ti)
OR
(('remote' OR 'technology based' OR 'mobile based' OR 'app based' OR 'wearab*') *AND* ('intervention*' OR 'program*' OR 'care' OR 'trial' OR 'consult*' OR 'support' OR 'service*' OR 'management' OR 'monitoring')):ti
OR
(('ambulatory' OR 'home' OR 'mobile technology') NEAR/4 'blood pressure' NEAR/4 'monitoring'):ti,ab,kw
OR
('mobile' NEAR/4 'application'):ti OR 'mobile app*':ti)

AND

('clinical trial'/exp OR 'intervention study'/exp OR 'cohort analysis'/exp OR 'program evaluation'/exp OR
('trial' OR 'trials' OR 'random*' OR 'controlled study' OR 'pilot study' OR 'cohort'):ab,ti,kw OR (('follow up' OR 'prospective' OR 'retrospective') AND 'study'):ab,ti,kw OR ('program*' OR 'intervention*'):ti)

AND

('Central America'/exp OR 'Hispanic'/exp OR 'South America'/exp OR 'Central America'/exp OR 'Caribbean'/exp OR ('low' NEAR/4 'middle' NEAR/4 'income' NEAR/4 'countries'):ti,ab,kw OR ('lower' NEAR/4 'middle' NEAR/4 'income' NEAR/4 'countries'):ti,ab,kw
OR
('latin*' OR 'hispanic*' OR 'south americ*' OR 'central americ*'):ab,ti,kw OR (('portuges*’ OR 'spanish') AND 'america*'):ti,ab,kw OR
('argentin*' OR 'bolivi*' OR 'brazil*' OR 'brasil*' OR 'chile' OR 'colombi*' OR 'costa ric*' OR 'cuba' OR 'cuban' OR 'dominican republic' OR 'ecuador*' OR 'el salvador*' OR 'guatemal*' OR 'hondura*’ OR 'mexic*' OR 'nicaragu*' OR 'panam*' OR 'paraguay*' OR 'peru*' OR 'puerto ric*' OR 'uruguay*' OR 'venezuel*' OR 'caribbean' OR 'aruba*' OR 'curacao*' OR 'sint maarten' OR 'saint martin' OR 'bonaire' OR 'sint eusta*' OR 'saint eusta*' OR 'saba' OR 'saban' OR 'antilles' OR 'caribbean' OR 'antigua' OR 'barbuda' OR 'bahamas' OR 'barbados' OR 'virgin islands' OR 'dominica' OR 'dominica' OR 'grenada' OR 'guadeloup*' OR 'haiti*' OR 'jamaica*' OR 'martinique' OR 'saint kitts' OR 'saint lucia' OR 'saint vincent' OR 'trinidad' OR 'trinidad' OR 'tobago'):ti,ab,kw,de,ff,ad)

NOT

((('child'/exp OR 'adolescent'/exp) NOT 'adult'/exp) OR 'conference abstract'/it OR (('child*':ti OR 'pediat*':ti OR 'paediat*':ti) NOT 'adult*':ti))

Web of Science (Web of Science Core Collection + SciELO Citation Index + Research Commons)

(TS=("hypertension" OR "prehypertension*" OR "diabet*" OR "dyslipidem*" OR "hyperlipid*" OR "elevated cholesterol" OR "high cholesterol" OR "glycemic control" OR "glycaemic control" OR "blood pressure" OR "lipid profile*" OR "hba1c" OR "blood glucose") OR TI=("cardiometabolic*" OR "metabolic"))

AND

(TI=("tele*" NOT ("television" OR "telephone"))

OR

TS=("teleconsult*" OR "telehealth" OR "telemedicine" OR "telemed*" OR "e consult*" OR "virtual consult*" OR "digital consult*" OR "online consult*" OR "internet consult*" OR "remote consult*" OR "video consult*" OR "digital intervention*" OR "mhealth" OR "m health" OR "ehealth" OR "e health" OR "e consult*" OR "e care" OR "e management" OR "technology enhanced" OR "text messag*")

OR

TS=(("remote" OR "online" OR "internet" OR "digi*" OR "tele" OR "virtual" OR "distance" OR "video") NEAR/4 ("consult*" OR "appointment*" OR "intervention*" OR "monitoring" OR "care" OR "management"))

OR

TS=(("remote" OR "online" OR "internet" OR "digi*" OR "tele" OR "virtual" OR "distance" OR "video") NEAR/4 "glycemic control")

OR

((TS=("online based" OR "webbased" OR "web based" OR "computer based" OR "internetbased" OR "video based") AND TI=("intervention*" OR "program*" OR "care" OR "trial" OR "consult*" OR "support" OR "service*" OR "management" OR "monitoring")))

OR

TI=(("remote" OR "technology based" OR "mobile based" OR "app based" OR "wearab*") AND ("intervention*" OR "program*" OR "care" OR "trial" OR "consult*" OR "support" OR "service*" OR "management" OR "monitoring"))

OR

TS=(("ambulatory" OR "home" OR "mobile technology") NEAR/4 "blood pressure" NEAR/4 "monitoring")

OR

TI=("mobile" NEAR/4 "application")

OR

TI=("mobile app*"))

AND

(TS=("trial" OR "trials" OR "random*" OR "controlled study" OR "clinical study" OR "pilot study" OR "cohort")

OR

TS=(("follow up" OR "prospective" OR "retrospective") AND "study")

OR

TI=("program*" OR "intervention*"))

AND

(TS=(("low" NEAR/4 "middle" NEAR/4 "income" NEAR/4 "countries") OR

("lower" NEAR/4 "middle" NEAR/4 "income" NEAR/4 "countries") OR

"latin*" OR "hispanic*" OR "south americ*" OR "central americ*" OR

(("portuges*" OR "spanish") AND "america*"))

OR

ALL=("argentin*" OR "bolivi*" OR "brazil*" OR "brasil*" OR "chile" OR "colombi*" OR "costa ric*" OR "cuba" OR "cuban" OR "dominican republic" OR "ecuador*" OR "el salvador*" OR "guatemal*" OR "hondura*" OR "mexic*" OR "nicaragu*" OR "panam*" OR "paraguay*" OR "peru*" OR "puerto ric*" OR "uruguay*" OR "venezuel*" OR "caribbean" OR "aruba*" OR "curacao*" OR "sint maarten" OR "saint martin" OR "bonaire" OR "sint eusta*" OR "saint eusta*" OR "saba" OR "saban" OR "antilles" OR "caribbean" OR "antigua" OR "barbuda" OR "bahamas" OR "barbados" OR "virgin islands" OR "dominica" OR "grenada" OR "guadeloup*" OR "haiti*" OR "jamaica*" OR "martinique" OR "saint kitts" OR "saint lucia" OR "saint vincent" OR "trinidad" OR "tobago"))

NOT

(TI=("child*" OR "pediat*" OR "paediat*") NOT TI=("adult*"))

AND

DT=(ARTICLE)

Cochrane Library (CENTRAL)

([mh Hypertension] OR [mh Prehypertension] OR [mh "Diabetes Mellitus"] OR [mh Dyslipidemias] OR [mh "Glycated Hemoglobin"] OR [mh ^"Blood Pressure"] OR [mh Cholesterol] OR [mh "Blood Glucose"] OR [mh "Glycemic Control"] OR [mh "Blood Pressure Monitoring, Ambulatory"]
OR
hypertension:ti,ab OR prehypertension*:ti,ab OR diabet*:ti,ab OR dyslipidem*:ti,ab OR hyperlipid*:ti,ab OR "elevated cholesterol":ti,ab OR "high cholesterol":ti,ab OR hypercholesterol*:ti,ab OR cardiometabolic*:ti OR metabolic:ti OR "glycemic control":ti,ab OR "glycaemic control":ti,ab OR "blood pressure":ti,ab OR ("lipid" NEXT profile*):ti,ab OR hba1c:ti,ab OR "blood glucose":ti,ab)

AND

([mh ^Telecommunications] OR [mh Telemedicine] OR [mh Telemetry] OR [mh Videoconferencing] OR [mh ^Telephone] OR [mh "Cell Phone"] OR [mh "Mobile Applications"]
OR
(tele*:ti NOT (television:ti OR telephone:ti)) OR teleconsult*:ti,ab OR telehealth:ti,ab OR telemedicine:ti,ab OR telemed*:ti,ab OR ("e" NEXT consult*):ti,ab OR ("virtual" NEXT consult*):ti,ab OR ("digital" NEXT consult*):ti,ab OR ("online" NEXT consult*):ti,ab OR ("internet" NEXT consult*):ti,ab OR ("remote" NEXT consult*):ti,ab OR ("video" NEXT consult*):ti,ab OR ("digital" NEXT intervention*):ti,ab OR mhealth:ti,ab OR "m health":ti,ab OR ehealth:ti,ab OR "e health":ti,ab OR ("e" NEXT consult*):ti,ab OR "e care":ti,ab OR "e management":ti,ab OR "technology enhanced":ti,ab OR ("text" NEXT messag*):ti,ab
OR

((remote OR online OR internet OR digi* OR tele OR virtual OR distance OR video) NEAR/4 (consult* OR appointment* OR intervention* OR monitoring OR care OR management)):ti,ab

OR

((remote OR online OR internet OR digi* OR tele OR virtual OR distance OR video) NEAR/4 (glycemic NEXT control)):ti,ab

OR

(((online NEXT based) OR webbased OR (web NEXT based) OR (computer NEXT based) OR (internetbased) OR (video NEXT based)):ti,ab AND (intervention* OR program* OR care OR trial OR consult* OR support OR service* OR management OR monitoring):ti)

OR

(((remote OR (technology NEXT based) OR (mobile NEXT based) OR (app NEXT based) OR wearab*) AND (intervention* OR program* OR care OR trial OR consult* OR support OR service* OR management OR monitoring))):ti

OR

(((ambulatory OR home OR (mobile NEXT technology)) NEAR/4 (blood NEXT pressure) NEAR/4 (monitoring))):ti,ab

OR

((mobile NEAR/4 application):ti OR (mobile NEXT app*):ti))

AND

([mh "Latin America"] OR [mh "Hispanic or Latino"] OR [mh "South America"] OR [mh "Central America"] OR [mh "Caribbean Region"] OR
(low NEAR/3 middle NEAR/3 income NEAR/3 countries):ti,ab OR (lower NEAR/3 middle NEAR/3 income NEAR/3 countries):ti,ab
OR
latin*:ti,ab OR latin* OR hispanic*:ti,ab OR hispanic* OR (south NEXT americ*):ti,ab OR (south NEXT americ*) OR (central NEXT americ*):ti,ab OR (central NEXT americ*) OR ((portuges*:ti,ab OR spanish:ti,ab) AND america*:ti,ab) OR ((portuges* OR spanish) AND america*) OR argentin*:ti,ab OR argentin* OR argentina OR bolivi*:ti,ab OR bolivi* OR bolivia OR brazil*:ti,ab OR brazil* OR brasil*:ti,ab OR brasil* OR brazil OR brasil OR chil*:ti,ab OR chil* OR chile OR colombi*:ti,ab OR colombi* OR colombia OR (costa NEXT ric*):ti,ab OR (costa NEXT ric*) OR costa rica:ti,ab OR costa rica OR cuba*:ti,ab OR cuba* OR cuba OR (dominican NEXT republic):ti,ab OR (dominican NEXT republic) OR dominican republic:ti,ab OR dominican republic OR ecuador*:ti,ab OR ecuador* OR ecuador OR (el NEXT salvador):ti,ab OR (el NEXT salvador) OR el salvador:ti,ab OR el salvador OR guatemal*:ti,ab OR guatemal* OR guatemala OR hondura*:ti,ab OR hondura* OR honduras OR mexic*:ti,ab OR mexic* OR mexico OR nicaragu*:ti,ab OR nicaragu* OR nicaragua OR panam*:ti,ab OR panam* OR panama OR paraguay*:ti,ab OR paraguay* OR paraguay OR peru*:ti,ab OR peru* OR peru OR (puerto NEXT ric*):ti,ab OR (puerto NEXT ric*) OR puerto rico:ti,ab OR puerto rico OR uruguay*:ti,ab OR uruguay* OR uruguay OR venezuel*:ti,ab OR venezuel* OR venezuela OR caribbean:ti,ab OR caribbean OR aruba:ti,ab OR aruba OR curacao:ti,ab OR curacao OR (sint NEXT maarten):ti,ab OR (sint NEXT maarten) OR sint maarten:ti,ab OR sint maarten OR (saint NEXT martin):ti,ab OR (saint NEXT martin) OR saint martin:ti,ab OR saint martin OR bonaire:ti,ab OR bonaire OR (sint NEXT eusta*):ti,ab OR (sint NEXT eusta*) OR sint eusta*:ti,ab OR sint eusta* OR (saint NEXT eusta*):ti,ab OR (saint NEXT eusta*) OR saint eusta*:ti,ab OR saint eusta* OR saba:ti,ab OR saba OR antilles:ti,ab OR antilles OR antigua:ti,ab OR antigua OR barbuda:ti,ab OR barbuda OR bahamas:ti,ab OR bahamas OR barbados:ti,ab OR barbados OR (virgin NEXT islands):ti,ab OR (virgin NEXT islands) OR virgin islands:ti,ab OR virgin islands OR dominica:ti,ab OR dominica OR grenada:ti,ab OR grenada OR guadeloupe:ti,ab OR guadeloupe OR haiti:ti,ab OR haiti OR jamaica:ti,ab OR jamaica OR martinique:ti,ab OR martinique OR (saint NEXT kitts):ti,ab OR (saint NEXT kitts) OR saint kitts:ti,ab OR saint kitts OR (saint NEXT lucia):ti,ab OR (saint NEXT lucia) OR saint lucia:ti,ab OR saint lucia OR (saint NEXT vincent):ti,ab OR (saint NEXT vincent) OR saint vincent:ti,ab OR saint vincent OR trinidad:ti,ab OR trinidad OR tobago:ti,ab OR tobago)

NOT
((([mh Child] OR [mh Adolescent]) NOT [mh Adult]) OR ((child*:ti OR pediat*:ti OR paediat*:ti) NOT adult*:ti))

Scopus

(TITLE-ABS-KEY(hypertension OR prehypertension* OR diabet* OR dyslipidem* OR hyperlipid* OR "elevated cholesterol" OR "high cholesterol" OR "glycemic control" OR "glycaemic control" OR "blood pressure" OR "lipid profile*" OR hba1c OR "blood glucose") OR TITLE(cardiometabolic* OR metabolic))

AND

( TITLE-ABS-KEY ( teleconsult* OR telehealth OR telemedicine OR telemed* OR "e consult*" OR "virtual consult*" OR "digital consult*" OR "online consult*" OR "internet consult*" OR "remote consult*" OR "video consult*" OR "digital intervention*" OR mhealth OR "m health" OR ehealth OR "e health" OR "e consult*" OR "e care" OR "e management" OR "technology enhanced" OR "text messag*" ) OR TITLE-ABS-KEY ( ( remote OR online OR internet OR digi* OR tele OR virtual OR distance OR video ) W/4 ( consult* OR appointment* OR intervention* OR monitoring OR care OR management ) ) OR TITLE-ABS-KEY ( ( remote OR online OR internet OR digi* OR tele OR virtual OR distance OR video ) W/4 "glycemic control" ) OR ( TITLE-ABS-KEY ( "online based" OR webbased OR "web based" OR "computer based" OR internetbased OR "video based" ) AND TITLE ( intervention* OR program* OR care OR trial OR consult* OR support OR service* OR management OR monitoring ) ) OR ( TITLE ( remote OR "technology based" OR "mobile based" OR "app based" OR wearab* ) AND TITLE ( intervention* OR program* OR care OR trial OR consult* OR support OR service* OR management OR monitoring ) ) OR TITLE-ABS-KEY ( ( "ambulatory" OR home OR "mobile technology" ) W/4 "blood pressure" W/4 monitoring ) OR TITLE ( mobile W/4 application ) OR TITLE ( "mobile app*" ) )

AND

( TITLE-ABS-KEY ( trial OR trials OR random* OR "controlled study" OR "clinical study" OR "pilot study" OR cohort )
OR
TITLE-ABS-KEY ( ( "follow up" OR prospective OR retrospective ) AND study )
OR
TITLE ( program* OR intervention* ) )

AND

( TITLE-ABS-KEY ( ( "low" W/4 "middle" W/4 "income" W/4 "countries" ) OR ( "lower" W/4 "middle" W/4 "income" W/4 "countries" ) OR latin* OR hispanic* OR "south americ*" OR "central americ*" OR ( ( portuges* OR spanish ) AND america* ) )
OR

TITLE-ABS-KEY(argentin* OR bolivi* OR brazil* OR brasil* OR chile OR colombi* OR "costa ric*" OR cuba OR cuban OR "dominican republic" OR ecuador* OR "el salvador*" OR guatemal* OR hondura* OR mexic* OR nicaragu* OR panam* OR paraguay* OR peru* OR "puerto ric*" OR uruguay* OR venezuel* OR caribbean OR aruba* OR curacao* OR "sint maarten" OR "saint martin" OR bonaire OR "sint eusta*" OR "saint eusta*" OR saba OR saban OR antilles OR caribbean OR antigua OR barbuda OR bahamas OR barbados OR "virgin islands" OR dominica OR grenada OR guadeloup* OR haiti* OR jamaica* OR martinique OR "saint kitts" OR "saint lucia" OR "saint vincent" OR trinidad OR tobago)
OR

AFFILCOUNTRY (argentin* OR bolivi* OR brazil* OR brasil* OR chile OR colombi* OR "costa ric*" OR cuba OR cuban OR "dominican republic" OR ecuador* OR "el salvador*" OR guatemal* OR hondura* OR mexic* OR nicaragu* OR panam* OR paraguay* OR peru* OR "puerto ric*" OR uruguay* OR venezuel* OR caribbean OR aruba* OR curacao* OR "sint maarten" OR "saint martin" OR bonaire OR "sint eusta*" OR "saint eusta*" OR saba OR saban OR antilles OR caribbean OR antigua OR barbuda OR bahamas OR barbados OR "virgin islands" OR dominica OR grenada OR guadeloup* OR haiti* OR jamaica* OR martinique OR "saint kitts" OR "saint lucia" OR "saint vincent" OR trinidad OR tobago))

AND NOT

(TITLE(child* OR pediat* OR paediat*) AND NOT TITLE(adult*))

AND ( LIMIT-TO ( DOCTYPE,"ar" ) )

1,806 records

Corrected search rerun on 8 September 2026, restricted to publications up to and including 2025. For verification, rerunning the originally executed syntax on 8 September 2026 retrieved 1,693 records; the corrected syntax retrieved 1,806 records (113 additional records).

LILACS

(("hypertension" OR "prehypertension*" OR "diabet*" OR "dyslipidem*" OR "cholesterol" OR "hyperlipid*" OR "cardiometabolic*" OR "metabolic"

OR "hipertension" OR "prehipertension*" OR "diabet*" OR "dislipidem*" OR "colesterol" OR "hiperlipid*" OR "cardiometabol*" OR "metabol*"

OR "hipertensão" OR "pré-hipertensão*" OR "diabet*" OR "dislipidem*" OR "colesterol" OR "hiperlipid*" OR "cardiometabólic*" OR "metabólic*"))

AND

(("tele*" OR "distance" OR "e consult*" OR "mhealth" OR "m health" OR "ehealth" OR "e health" OR "e consult*" OR "e care" OR "e management" OR "technology enhanced" OR "text messag*" OR "mobile app*" OR "wearab*" OR "online based" OR "webbased" OR "web based" OR "computer based" OR "internetbased" OR "video based" OR "remote" OR "online" OR "internet" OR "digi*" OR "tele" OR "virtual" OR "distance" OR "video" OR "ambulatory monitoring" OR "home monitoring" OR "mobile technology"

OR "tele*" OR "distancia" OR "consulta electrónica*" OR "telemedicina" OR "teleconsulta*" OR "salud electrónica*" OR "mensaj* de texto" OR "aplicación móvil*" OR "ponible*" OR "en línea" OR "basado en web" OR "computadora" OR "base de internet" OR "remoto" OR "virtual" OR "monitoreo ambulatorio" OR "monitoreo en casa" OR "tecnología móvil"

OR "tele*" OR "distância" OR "consulta eletrônica*" OR "telemedicina" OR "teleconsulta*" OR "saúde eletrônica*" OR "mensagem de texto" OR "aplicativo móvel*" OR "vestível*" OR "online" OR "baseado na web" OR "computador" OR "base de internet" OR "remoto" OR "virtual" OR "monitoramento ambulatorial" OR "monitoramento domiciliar" OR "tecnologia móvel"))

AND

(("trial" OR "trials" OR "random*" OR "controlled study" OR "clinical study" OR "pilot study" OR "cohort" OR "follow up" OR "prospective" OR "retrospective" OR "program*" OR "intervention*"

OR "ensayo" OR "ensayos" OR "aleatoriz*" OR "estudio controlado" OR "estudio clínico" OR "estudio piloto" OR "cohorte" OR "seguimiento" OR "prospectivo" OR "retrospectivo" OR "programa*" OR "intervención*"

OR "ensaio" OR "ensaios" OR "aleatório*" OR "estudo controlado" OR "estudo clínico" OR "estudo piloto" OR "coorte" OR "acompanhamento" OR "prospectivo" OR "retrospectivo" OR "programa*" OR "intervenção*")

AND NOT (("child*" OR "pediat*" OR "paediat*") AND NOT "adult*"))
