# Supplementary material for: Telemedicine for cardiometabolic disease control in Latin America and the Caribbean: A systematic review and meta-analysis
Source: Prev Med Rep. 2026 Sep 23;71:103633. doi: 10.1016/j.pmedr.2026.103633 (PMC13634337; doi:10.1016/j.pmedr.2026.103633)
Supplement: Supplementary file 2 [file mmc9.docx]

**Supplementary References – Studies Included in the Systematic Review**

S1. Vázquez-Martínez VH, Martínez-Bautista H, Loera-Morales J, Garzón Garcia FD, Velez-Maldonado D, Muñoz-Villegas P. Use of telephone calls to manage glycemic control at Mexico's northern border. Front Endocrinol (Lausanne) 2025; 16: 1420244.

S2. Piette JD, Mendoza-Avelares MO, Ganser M, Mohamed M, Marinec N, Krishnan S. A preliminary study of a cloud-computing model for chronic illness self-care support in an underdeveloped country. Am J Prev Med 2011; 40: 629-32.

S3. Urbina-Arronte LE, Alcántara-Garcés MT, Ochoa Ramírez A, Velázquez-Jurado HR, García-Ulloa AC, Hernández-Jiménez S. Metabolic, self-care and mental health characteristics of patients with type 2 diabetes in a hybrid (face-to-face and virtual) comprehensive care program. Ther Adv Endocrinol Metab 2025; 16: 20420188251367234.

S4. Debon R, Bellei EA, Biduski D, et al. Effects of using a mobile health application on the health conditions of patients with arterial hypertension: a pilot trial in the context of Brazil's Family Health Strategy. Sci Rep 2020; 10: 6009.

S5. Rodríguez-Montes OE, Gogeascoechea-Trejo MDC, Bermúdez-Tamayo C. Efficacy of a mobile application for glycemic control in type 2 diabetes: a randomized clinical trial. Diabetes Epidemiol Manag 2025; 19: 100279.

S6. Esandi ME, Ortiz Z, Bernabei V, et al. Evaluating the implementation of a hypertension program based on mHealth and community pharmacies integration to primary care centers at a municipality level in Argentina during the COVID-19 pandemic. Front Health Serv 2024; 4: 1263331.

S7. Moreira AM, Rados DV, de Farias CB, et al. Effects of nurse tele support via telephone calls on transition between specialized and primary care in type 2 diabetes mellitus patients: a CONSORT-compliant randomized clinical trial. Endocrine 2025; 87: 978-86.

S8. Cruz Gutiérrez CM. Efectividad de la telemedicina en el control del nivel de la hemoglobina glicosilada en pacientes con diabetes mellitus 2 [thesis]. Trujillo: Universidad Privada Antenor Orrego; 2023.

S9. Rodrigues DLG, Belber GS, Padilha FVDQ, et al. Teleconsultation on patients with type 2 diabetes in the Brazilian public health system: a randomised, pragmatic, open-label, phase 2, non-inferiority trial (TELECONSULTA diabetes trial). Lancet Reg Health Am 2024; 39: 100923.

S10. Franco DW, Alessi J, de Carvalho TR, et al. The impact of a telemedicine intervention on the metabolic profile of diabetes mellitus patients during the COVID-19 pandemic: a randomized clinical trial. Prim Care Diabetes 2022; 16: 745-52.

S11. Ramos-Zavala MG, Grover-Paez F, Cardona-Munoz EG, et al. Comparison of the use of blood pressure telemonitoring versus standard medical care in the achievement of short-term therapeutic goals in blood pressure in patients with uncontrolled hypertension: an open-label clinical trial. JRSM Cardiovasc Dis 2023; 12: 20480040231178585.

S12. Ciuffarella C, Maloberti A, Quarti-Trevano F, Dell'Oro R, Facchetti R, Grassi G. Results of a telemedicine program in patients with cardiovascular risk factors in low- and middle-income countries. High Blood Press Cardiovasc Prev 2024; 31: 513-5.

S13. Calderón-Anyosa R, Tincopa JP, Raza M, Cárcamo CP. Randomized controlled trial of home telemonitoring of blood pressure with an adapted tensiometer with SMS capability. Eur J Investig Health Psychol Educ 2023; 13: 440-9.

S14. Spósito P, Taborda A, Adano A, et al. Telemedicina en el control de la hipertensión arterial. Rev Urug Med Interna 2023; 8: 38-52.

S15. Aguiar PM, da Silva CHP, Chiann C, Dórea EL, Lyra Junior DP, Storpírtis S. Pharmacist-physician collaborative care model for patients with uncontrolled type 2 diabetes in Brazil: results from a randomized controlled trial. J Eval Clin Pract 2018; 24: 22-30.

S16. Gómez AM, Henao DC, León-Vargas F, et al. Efficacy of the mHealth application in patients with type 2 diabetes transitioning from inpatient to outpatient care: a randomized controlled clinical trial. Diabetes Res Clin Pract 2022; 189: 109948.

S17. Anzaldo-Campos MC, Contreras S, Vargas-Ojeda A, Menchaca-Díaz R, Fortmann A, Philis-Tsimikas A. Dulce Wireless Tijuana: a randomized control trial evaluating the impact of Project Dulce and short-term mobile technology on glycemic control in a family medicine clinic in Northern Mexico. Diabetes Technol Ther 2016; 18: 240-51.

S18. Lange I, Campos-Romero S, Urrutia-Bunster M, et al. Efecto de un modelo de apoyo telefónico en el auto-manejo y control metabólico de la diabetes tipo 2, en un centro de atención primaria, Santiago, Chile. Rev Med Chil 2010; 138: 729-37.

S19. Díaz Rueda E, Ortiz Suarez C. Tecnologías de la información y comunicación en hipertensión arterial: ensayo clínico controlado. Rev Cienc Cuidad 2018; 15: 155-68.

S20. Piette JD, Datwani H, Gaudioso S, et al. Hypertension management using mobile technology and home blood pressure monitoring: results of a randomized trial in two low/middle-income countries. Telemed J E Health 2012; 18: 613-20.

S21. Kim JK, Jo HY, Malo MA, Nam EW. Effectiveness of a comprehensive blood pressure control program in primary health care in Peru. Rev Panam Salud Publica 2020; 44: e18.

S22. Khan R, Gopaulsingh J, Gopee Z, et al. The effectiveness of telemedicine in the management of chronic diseases (diabetes, hypertension, and coronary heart disease) at primary care facilities in Eastern Trinidad during the COVID-19 pandemic. Telemed J E Health 2024; 30: e2311-8.
